# Surgical repair of unusual variant of double-outlet right ventricle with discordant atrioventricular connections and superior-inferior ventricles

**DOI:** 10.1016/j.xjtc.2023.12.004

**Published:** 2023-12-29

**Authors:** Hamood Al Kindi, Pranav Kandachar, Abdullah Mohsen, Abdullah A. Balushi, Tuqa Al Lawati, Madan Mohan Maddali, Robert Henry Anderson

**Affiliations:** a Division of Cardiothoracic Surgery, Department of Surgery, Sultan Qaboos University Hospital, Seeb, Oman; b Department of Cardiothoracic Surgery, National Heart Center, The Royal Hospital, Muscat, Oman; c Department of Pediatric Cardiology, National Heart Center, The Royal Hospital, Muscat, Oman; d Department of Cardiac Anesthesia, National Heart Center, The Royal Hospital, Muscat, Oman; e Institute of Genetic Medicine, Newcastle University, Newcastle upon Tyne, United Kingdom

**Figure:**
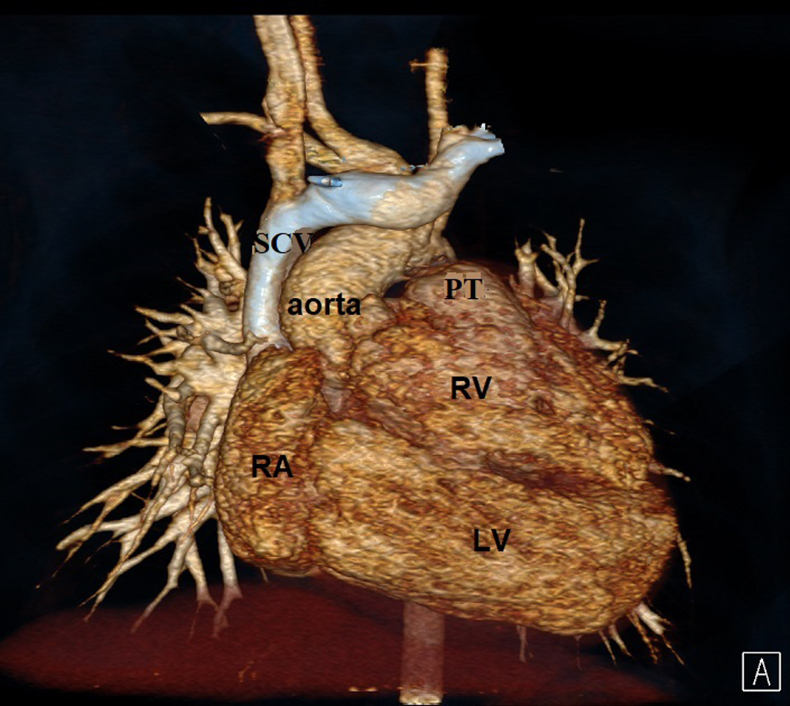
CT 3D reconstruction demonstrating the anatomy of the superior inferior ventricles.

**Central Message**

The surgical management of double-outlet right ventricle and superior-inferior ventricles is challenging. A personalized approach facilitates the planning for successful biventricular repair.

The term “superior-inferior ventricles” represents the rare positioning of the ventricles one above the other, rather than side-by-side.^1^^,^^2^ We report the successful surgical repair in a child with superior-inferior ventricles, double-outlet right ventricle with discordant atrioventricular connections, and with the aortic root in an unusual right-sided position (institutional review board no. MoH/CSR/CR#2023/37, November 16, 2023). The parents of the patients provided informed written consent for publication of study data.

## Case Report

**Figure 1:**
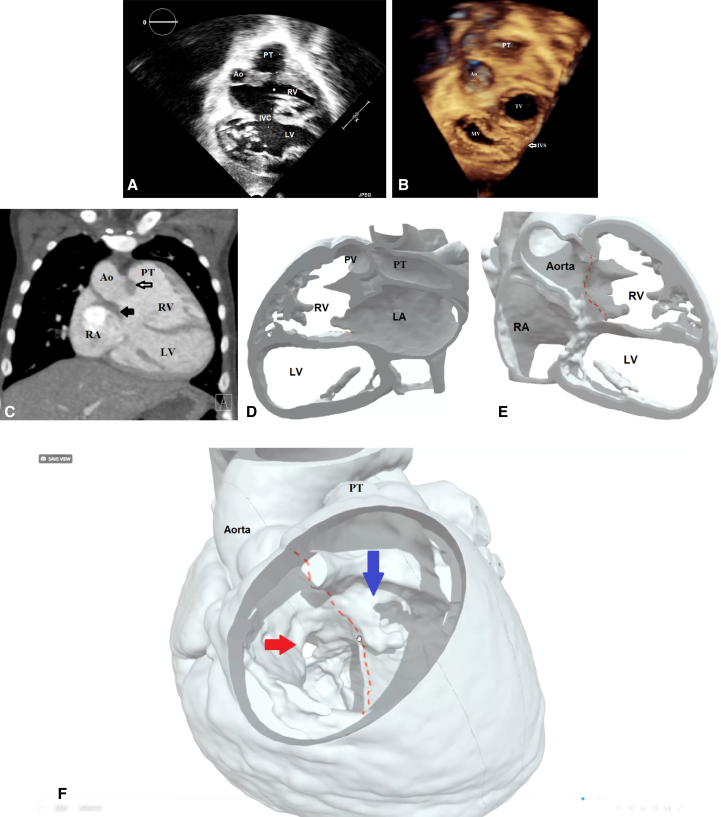
A, Two-dimensional transthoracic echocardiogram showing the horizontal septum and the location of the interventricular communication, the great arteries, and the ventricular relationship. B, Three-dimensional (3D) echocardiogram showing the relationship of the atrioventricular and the arterial valves. C, Computed tomography scan (coronal view) illustrates the relationship of the great arteries, the atrioventricular valves, the location of the interventricular communication, and the bilateral infundibula. *White arrow*: the outlet septum; *black arrow*: the infundibulum separating the aortic root from the mitral valve. D, 3D modeling for preoperative surgical planning. The left atrium (*LA*) is connected to the superior right ventricle (RV), which is connected the pulmonary trunk. E, Virtual cut in the RVOT to demonstrate the location of the interventricular communication in relationship with ventricles, great arteries, and the AV valves. *Red lines* indicate the planned suture line for the intraventricular tunnel. F, 3D model: The right atrium (*RA*) is connected to the left ventricle (LV). The aorta is arising from the RV; however, it is facing the LV through the interventricular communication. *Red lines* indicate the planned suture line for creation of the intraventricular tunnel. *Red arrow* is the mitral inflow to the inferior left ventricle, and the *blue arrow* is the tricuspid inflow to the superior right ventricle. *Ao*, Aorta; *PT*, pulmonary trunk; *RV*, right ventricle; *IVC*, interventricular communication; *LV*, left ventricle; *TV*, tricuspid valve; *MV*, mitral valve; *IVS*, interventricular septum; *PV*, pulmonary valve.

A newborn girl with complex congenital anomalies was referred to our center for evaluation. Cross-sectional and 3D transthoracic echocardiography showed normal atrial and venous connections, discordant atrioventricular connections, and double-outlet right ventricle (Figure 1, *A* and *B*). A superior and inferior relationship was observed between the ventricles. The ventricular septum was almost horizontal, with a large interventricular communication. The aortic root was positioned on the right side, despite the left-handed ventricular topology. A muscular infundibulum supported each arterial root, and the interventricular communication was below the aortic valve. The neonate underwent ligation of a persistently patent arterial duct and banding of the pulmonary trunk, as palliative measures. The patient remained stable and continued to thrive without any complications.

**Figure 2:**
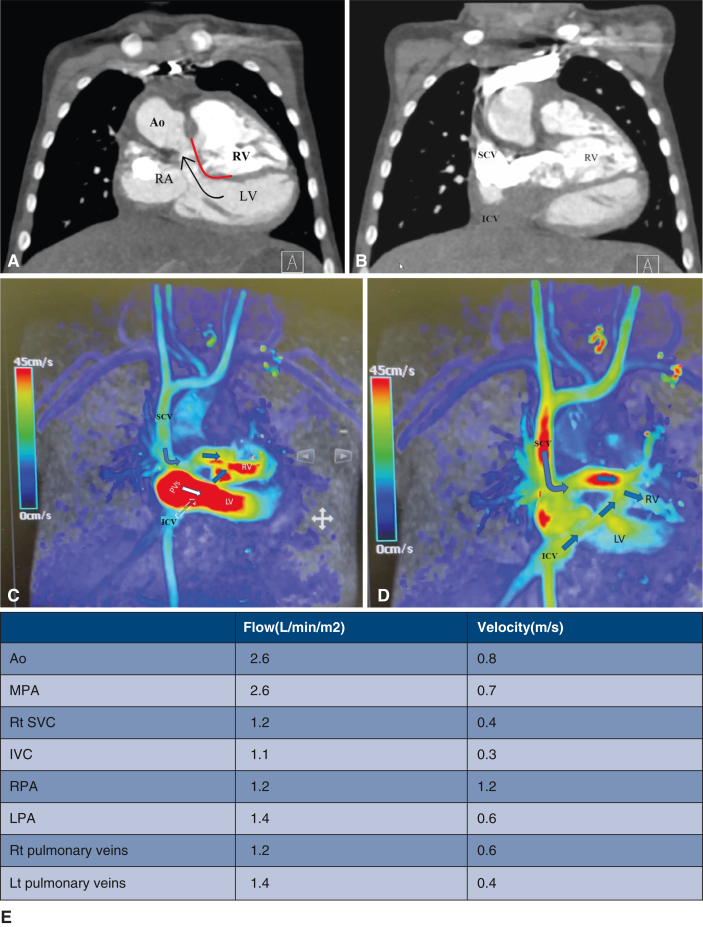
A, Postoperative computed tomography (CT) scan illustrates the outflow of the left ventricle and the right ventricle. The *red line* marks the intraventricular tunnel. The *black arrow* indicates the direction of flow in the left ventricular outflow tract. B, Postoperative CT scan illustrates the systemic venous baffle to the right ventricle. C and D, Qualitative assessment of the systemic and pulmonary venous pathway using 4-dimensional flow magnetic resonance imaging. E, Quantitative assessment of the flow and velocity using 4-dimensional flow magnetic resonance imaging. *Ao*, Aorta; *RV*, right ventricle; *RA*, right atrium; *LV*, left ventricle; *SCV*, superior caval vein; *ICV*, inferior caval vein; *MPA*, main pulmonary artery; *Rt*, right; *RPA*, right pulmonary artery; *LPA*, left pulmonary artery; *Lt*, left.

At the age of 20 months, cardiac catheterization showed suitable hemodynamics for intracardiac repair. To assist with surgical planning, a contrast computed tomographic scan was performed, along with 3-dimensional (3D) modeling and printing (Figure 1, *C*-*F*). The surgical repair (Video 1) was done on hypothermic cardiopulmonary bypass under cardioplegic arrest. The brachiocephalic and inferior caval veins were cannulated for venous access, whereas the distal ascending aorta was the site of arterial cannulation. The outlet of the morphologically right ventricle was opened, and an intraventricular tunnel was created between the interventricular communication and the aortic root using a large bovine pericardial patch. Pledgetted mattress sutures were placed within the ventriculo-infundibular fold in the right ventricle, and parallel sutures were used on the area of fibrous continuity between the mitral and tricuspid valve leaflets. The suture line was then transitioned along the outlet septum and finally to the free parietal wall of the right ventricle. A bovine pericardial patch was used to close the right ventricular incision and reconstruct the pulmonary trunk. The right atrium was opened, the interatrial septum was excised, and the coronary sinus was unroofed as part of an atrial switch using the Mustard technique. The child was easily separated from cardiopulmonary bypass in sinus rhythm with minimal inotropic support. The child was discharged on the twelfth day after a smooth postoperative course. Postoperative echocardiography at the 2-month follow-up revealed unobstructed pulmonary and systemic venous pathways, with no obstruction in either ventricular outflow tract, and good biventricular function (Figure 2).

## Comment

The interventricular septum lies horizontally in hearts with superior-inferior ventricles, unlike the twisting of the ventricular mass seen in a “criss-cross heart” due to rotation of the atrioventricular connections.^2^ Various surgical approaches have been reported for individuals with this finding, depending on the different segmental combination.^3^^,^^4^ When double-outlet right ventricle is found with discordant atrioventricular connections and left-handed ventricular topology, the aortic root is usually anterior and left-sided, usually with a subpulmonary interventricular communication. In our patient, the aortic root was located on the right side and could be tunneled directly to the morphologically left ventricle through the interventricular defect. It was then necessary also to perform an atrial redirection procedure. This type of double-outlet right ventricle is similar to the situation when the atrioventricular connections are discordant but with concordant ventriculoarterial connections and side-by-sided outflow tracts. The latter combination produces “transposition” physiology, for which simple atrial redirection is the optimal surgical procedure.^5^^,^^E1^^,^^E2^ When contemplating biventricular repair for double-outlet right ventricle, the important factors to consider are the size of the right ventricle, and the location of the interventricular communication. The right ventricle in our ventricle was small but tripartite, and the z score for the tricuspid valve was normal. In a similar patient with double-outlet right ventricle, discordant atrioventricular connections, and superior-inferior ventricles an arterial switch was performed along with the Senning procedure.^3^ In that patient, the interventricular communication was subpulmonary area. It was the unexpected arrangement of the outflow tracts that underscored our approach. By tunneling the interventricular communication to the right-sided aortic root, we were able to avoid performing the arterial switch procedure. The arrangement is indicative of disharmony between the topology of the ventricular and arterial segments.

Tailored surgical approaches have become more feasible with advanced imaging and 3D printing.^E3^^,^^E4^ In our patient, 3D printing helped us understand the segmental relationships and confirm the feasibility of tunneling the interventricular communication to the aortic root. Four-dimensional flow magnetic resonance imaging is a valuable modality for assessing blood flow velocities, wall shear stress, and potential baffle leaks and outflow tract obstructions.^E5^

## Conflict of Interest Statement

The authors reported no conflicts of interest.

The *Journal* policy requires editors and reviewers to disclose conflicts of interest and to decline handling or reviewing manuscripts for which they may have a conflict of interest. The editors and reviewers of this article have no conflicts of interest.

## Supplementary Data

Video 1 Surgical repair of superoinferior ventricles with double-outlet right ventricle and discordant atrioventricular connections. The video also includes the preoperative imaging and surgical planning using 3D modeling and the postoperative CT scan, and 4D-flow MRI. Video available at: https://www.jtcvs.org/article/S2666-2507(23)00477-7/fulltext.
