# Supplementary figures and images for: Surgical repair of unusual variant of double-outlet right ventricle with discordant atrioventricular connections and superior-inferior ventricles
Source: JTCVS Tech. 2023 Dec 29;24:145–9. doi: 10.1016/j.xjtc.2023.12.004 (PMC11145076; doi:10.1016/j.xjtc.2023.12.004)

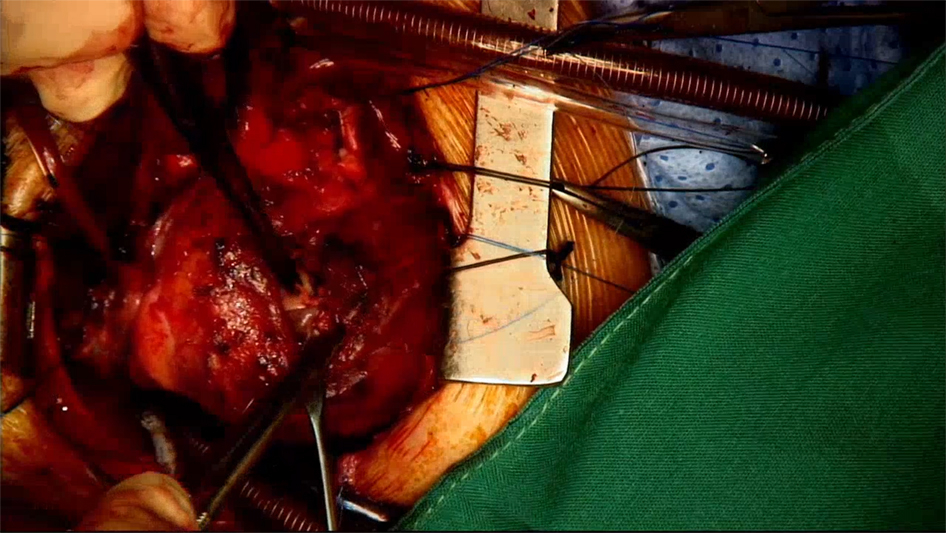

Supplement: Video 1 — Surgical repair of superoinferior ventricles with double-outlet right ventricle and discordant atrioventricular connections. The video also includes the preoperative imaging and surgical planning using 3D modeling and the postoperative CT scan, and 4D-flow MRI. Video available at: https://www.jtcvs.org/article/S2666-2507(23)00477-7/fulltext. [file fx2.jpg]
